# Relative Age Effects in Basketball: Exploring the Selection into and Successful Transition Out of a National Talent Pathway

**DOI:** 10.3390/sports9070101

**Published:** 2021-07-12

**Authors:** Adam L. Kelly, Sergio Lorenzo Jiménez Sáiz, Alberto Lorenzo Calvo, Alfonso de la Rubia, Daniel T. Jackson, Mark A. Jeffreys, Charlie Ford, Dave Owen, Sara Diana Leal dos Santos

**Affiliations:** 1 Research Centre for Life and Sport Sciences (CLaSS), Department of Sport and Exercise, School of Health Sciences, Birmingham City University, Birmingham B15 3TN, West Midlands, UK; Daniel.Jackson6@mail.bcu.ac.uk (D.T.J.); Mark.Jeffreys@bcu.ac.uk (M.A.J.); 2 Centre for Sport Studies, Universidad Rey Juan Carlos, Fuenlabrada, 28933 Madrid, Spain; sergio.jimenez.saiz@urjc.es; 3 Departamento de Deportes, Facultad de Ciencias de la Actividad Física y del Deporte—INEF, Universidad Politécnica de Madrid, 28040 Madrid, Spain; alberto.lorenzo@upm.es (A.L.C.); alfonso.delarubia@upm.es (A.d.l.R.); 4 Basketball England, NCS, Gate 13, Etihad Campus, Rowsley Street, Manchester M11 3FF, Lancashire, UK; charlie.ford@englandboxing.org (C.F.); dave.owen@basketballengland.co.uk (D.O.); 5 Research Center in Sports Sciences, Health Sciences and Human Development (CIDESD), University of Trás-os-Montes and Alto Douro, 5000-801 Vila Real, Portugal; sarasantos_8@hotmail.com

**Keywords:** talent identification, talent development, athlete development, expertise, sports coaching, growth and maturation

## Abstract

Relative age effects (RAEs) appear consistently prevalent throughout the youth basketball literature. However, the selection into and successful transition out of a national talent pathway in basketball is yet to be explored. Thus, the primary aim of this study was to explore the influence of relative age, gender, and playing time based on the selection into the Regional Talent Hubs and Basketball England youth teams (U16, U18, and U20) and the successful transition into the England National Senior Teams. Participants who were selected into the male (*n* = 450) and female (*n* = 314) Basketball England Talent Pathway were allocated into one of three cohorts: (a) Regional Talent Hubs (U12 to U15; *n* = 183), (b) England National Youth Teams (U16, U18, and U20; *n* = 537), and (c) England National Senior Teams (*n* = 44). A chi-square test was used to compare the birth quarter (BQ) distributions of each cohort against the expected distributions, with a Cramer’s V (Vc) used to interpret effect sizes. Odds ratios (OR) and 95% confidence intervals were also calculated to compare the likelihood of each BQ being represented. Males revealed significant RAEs across both the Regional Talent Hubs (*p* < 0.001, Vc > 0.29, OR = 10) and England National Youth Teams (*p* < 0.001, Vc > 0.17, OR = 3.1). In comparison, females only had significant RAEs in the Regional Talent Hubs (*p* < 0.001, Vc > 0.29, OR = 2.3). Despite RAEs being prevalent throughout youth levels, there were no significant differences in the BQ distribution based on playing time and those who made the successful transition to the England National Senior Teams. These findings demonstrate the potential mechanisms of RAEs in basketball, as well as the impetus to explore more equitable competition structures within the England Basketball Talent Pathway.

## 1. Introduction

Achieving expertise in basketball is a complex and multidimensional process [1]. For instance, a diverse sporting background during childhood [2], high jump and fast sprint capabilities [3], and advanced achievement and competitiveness motivation [4] have all been revealed as contributing factors towards greater long-term player development in basketball [5]. However, despite these multiple factors, senior professional and international basketball players must often acquire one common characteristic if they are to be selected: being tall [6]. For example, although the national average height for an American adult male is 5 ft 9 in (180 cm; [7]), the average height for a male professional basketball player competing in the National Basketball Association (NBA) is 6 ft 6 in (201 cm; [8]). Similarly, females competing in the Women’s NBA (WNBA) are on average taller (6 ft; 183 cm; [9]) when compared with the average American adult female national norm (5 ft 4 in; 165 cm; [7]). Taller players can have an advantage in basketball due to their shots travelling less distance to the basket, they start closer to the rebound, and their ability to reach higher into the air offers a greater opportunity of blocking shorter players’ passes and shots [10]. Since being taller can offer advantages in senior professional and international basketball competitions, it may also have important implications on talent identification and development processes in youth basketball [6].

Common selection and participation biases in youth sport are based on relative age effects—RAEs [11]. Relative age effects are based on a concept that demonstrates how youth athletes who are born at the beginning of a (bi)annual age group (e.g., under-16, under-18, and under-20) are more likely to be selected and/or participate in youth sport compared with their relatively younger peers [12]. For example, in their systematic review of 57 studies across a range of sports, Smith et al. [13] revealed 25% more female athletes born in the first birth quarter (BQ1; January, February, and March) of an annual age group were selected and/or participated in youth sport compared with those born in the last BQ (BQ4; October, November, and December). A possible explanation for this overrepresentation of relatively older athletes is due to the physiological and psychosocial advantages of being born earlier [14]. In basketball, for instance, height, body mass, running speed, and explosive power are important factors for greater performance [15], and these can be greatly affected by relative age [16]. In a systematic review of nine studies, de la Rubia et al. [17] demonstrated that RAEs had a significant impact on basketball performance, particularly within males and at formative ages (14–18 years). Since there appears to be a reliance on advanced physiological and anthropometrical characteristics for greater selection opportunities and performance levels in basketball, these factors may fortuitously exacerbate RAEs in youth settings [18].

Pronounced RAEs in youth basketball have been previously reported in various mixed-gender case studies from Brazil [19,20], France [21], Germany [22], Japan [23], North America [24], Poland [25], Portugal [26], and Spain [27]. The prevalence of RAEs has also been revealed in international competitions, such as the Olympic Games [28], World Championships [29], European Championships [30], and the Adidas Next Generation Tournament (the top European competition for under-18s; [31]). As an example, Arrieta et al. [32] found a significant overrepresentation of those born earlier in the selection year, as well as an association between relatively older age and performance outcomes (e.g., increased playing time), during the under-16, under-18, and under-20 International Basketball Federation (FIBA) European Championships. Further research has suggested competition level, age, and gender are important considerations when examining who is at risk of RAEs. For instance, García et al. [33] found weaker RAEs in the female youth FIBA World Championships compared with their male equivalents. Furthermore, they illustrated how RAEs decreased with older age, whereby they were highest in the under-17 competition, slightly less but also significant in the under-19 competition, and insignificant in the under-21 competition. Together, these findings highlight the importance of exploring a nation-specific context, as well as considering the influence of competition level, age, gender, and performance outcomes as contributing sources of RAEs in youth basketball.

Although RAEs are common throughout youth sport and especially in team sports, findings at senior levels appear mixed. On the one hand, some research in basketball has reported *knock-on effects* of RAEs during adulthood [34]. For instance, López de Subijana and Lorenzo [27] revealed long-term success at the senior professional level in Spanish basketball (e.g., BQ1 30% vs. BQ4 19%) was due to a relative age bias in selection at youth level (e.g., BQ1 38% vs. 9%). On the other hand, however, some literature has highlighted no RAEs at the senior professional level when compared with their youth cohorts (e.g., [35]). In the context of basketball, *no RAEs* were demonstrated at the senior international level in athletes who participated in the Olympic Games of London 2012 [36], and Rio de Janeiro 2016 [28]. In fact, *reversal effects* of relative age (e.g., [37]) or an *underdog hypothesis* [38] have been reported during the transition from youth levels to senior status in some team sports. For example, Kelly et al., [39] demonstrated potential late birthday benefits in soccer through the lens of the underdog hypothesis, whereby those born in BQ4 were approximately four times more likely to achieve a professional contract once selected into a youth academy compared with those born in BQ1. In sum, the variabilities in the outcomes at the senior level suggest RAEs may be associated with a combination of socio-environmental factors and sport-specific performance demands [40]. Interestingly, however, it appears the transition from youth level to senior representation in international basketball is yet to be explored. In doing so, it may offer further evidence of RAEs, while also depicting the effectiveness of existing talent identification and development processes.

Basketball England has participated in male and female international youth competitions at under-16, under-18, and under-20 age groups for over two decades. The main aim of these national youth teams is to develop and prepare young players for the senior Basketball England teams [41]. More recently, Basketball England created a pool of ten Regional Talent Hubs as an entry level into their Talent Pathway. The main purpose of these Talent Hubs is to identify talented young players aged 11 to 15 years, and offer them the greatest opportunity to develop towards the national teams in the future [42]. Despite the range of literature exploring the talent identification and development processes in youth basketball, the selection into and successful transition out of a national talent pathway in youth basketball is yet to be explored across both male and female cohorts. As such, the first aim of this current study was to explore the BQ distribution of the male and female Regional Talent Hubs (i.e., under-12 to under-15) as the entry level to the Basketball England Talent Pathway. Next, the BQ distribution of the male and female England National Youth Teams (i.e., under-16, under-18, and under-20) was analysed. Further analysis explored the BQ distribution of those who successfully transitioned from these England National Youth Teams to the male and female England National Senior Teams. Lastly, to test the competition opportunities once participants were selected for the England National Youth Teams and England National Senior Teams, differences between the average number of minutes played per game between BQs were compared. Based on the pre-existing literature, it was hypothesised that there would be an overrepresentation of relatively older players who played more minutes at youth levels. In comparison, it was hypothesised that there would be no RAEs at senior levels.

## 2. Methods

### 2.1. Sample and Procedures

The sample comprised of 764 participants who were selected into the male (*n* = 450) and female (*n* = 314) Basketball England Talent Pathway. Participants were allocated into one of three mixed-gender cohorts based on their playing level: (a) Regional Talent Hubs (under-12 to under-15; *n* = 183), (b) England National Youth Teams (under-16, under-18, and under-20; *n* = 537), and (c) England National Senior Teams (*n* = 44). Prior to 2016, the home nations of England, Scotland, and Wales held FIBA licences individually and competed in European competitions as England, Scotland, or Wales. As a result of an Olympic legacy agreement, the three home nations gave up their individual licences with Great Britain Basketball holding the licence for senior competition, which accordingly meant that the under-16, under-18, and under-20 teams came together and competed internationally as Great Britain rather than individual nations (i.e., England). Thus, those selected after 2016 may have represented Great Britain rather than England at youth and senior levels in certain competitions (e.g., Olympic Games), although Basketball England still competes in other competitions (e.g., Commonwealth Games). Male and female cohorts were also analysed independently in order to investigate gender-specific contexts. Participants from the Regional Talent Hubs were registered during the last three seasons since their inception (2017/18, 2018/19, and 2019/20). Participants from the England National Youth Teams were selected for the under-16, under-18, and/or under-20 age groups during the last 20 years (2000 to 2020) since data collection began. Participants from the England National Senior Teams only included those who were previously selected for the England National Youth Teams in order to explore the selection into and successful transition out of a national youth development programme. Data were provided to the research team by Basketball England in an attempt to better understand their existing organisational structures, which is part of an ongoing collaboration.

This methodology divided the year into four three-month BQs in accordance with each respective cohort’s selection cut-off date. Thus, international regulation age group cut-off dates were applied, with the 1st of January as *month 1* and 31st December as *month 12* (e.g., [43]). Each participant was subsequently assigned a BQ corresponding to their birthdate to create an observed BQ distribution within each of the cohorts. The observed BQ distributions from each cohort were compared against the expected BQ distribution calculated from average national live births (i.e., National Norms applied from the Office for National Statistics [ONS], 2015). In addition, the average number of minutes played across the England National Youth Teams and England National Senior Teams were analysed to test the competition opportunities between BQs. This study was ethically approved at both organisational (Basketball England) and institutional (Birmingham City University) levels.

### 2.2. Data Analysis

A chi-square (χ^2^) goodness of fit test was used to compare the BQ distributions of each cohort against the expected BQ distributions (ONS, 2015), following procedures outlined by McHugh [44]. Since this test does not reveal the magnitude of difference between the BQ distributions for significant χ^2^ outputs, a Cramer’s V (*V*c) was also used. The *V*c was interpreted as per conventional thresholds for correlation, whereby a value of 0.06 or more indicated a small effect size, 0.17 or more indicated a medium effect size, and 0.29 or more indicated a large effect size [45]. Odds ratios (ORs) and 95% confidence intervals (CIs) were calculated in order to compare the likelihood of each BQ being represented (CIs including one marked no association). In addition, the differences between the average number of minutes played per game across each BQ were analysed using a one-way ANOVA. Results were considered significant for *p* < 0.05.

## 3. Results

**Figure 1:**
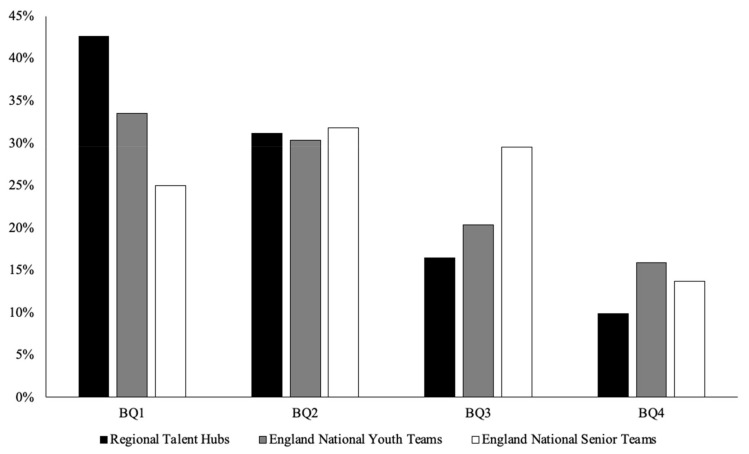
Regional Talent Hubs, England National Youth Teams, and England National Senior Teams BQ distributions.

There was a significant difference between the BQ distributions of the Regional Talent Hubs compared with the National Norms for the male, female, and combined cohorts (*p* < 0.001), with large effect sizes (*V*c > 0.29). The ORs showed an increased likelihood of relatively older players being selected, with the highest OR being BQ1 vs. BQ4 (ranging from 2.27 to 9.98). Similarly, there was a significant difference between the BQ distributions of the England National Youth Teams compared with the National Norms for the male and combined cohorts (*p* < 0.001), with medium effect sizes (*V*c > 0.17). The ORs showed an increased likelihood of relatively older players being selected, with the highest OR being BQ1 vs. BQ4 (ranging from 2.11 to 3.07). However, there were no significant differences between the BQ distributions of England National Youth Teams compared with the National Norms for the female cohort (*p* = 0.153). Moreover, there were no significant differences between the BQ distributions of the England National Senior Teams compared with the National Norms for the male, female, and combined cohorts (*p* = 0.35). Table 1 and Figure 1 show the χ^2^ analysis and BQ distributions, respectively. Lastly, the ANOVAs for the England National Youth Teams (*F*(_1.529_) = 0.071, *p* = 0.79) and the England National Senior Teams (*F*(_1.42_) = 0.095, *p* = 0.76) revealed no significant differences between the average number of minutes played across BQs.

## 4. Discussion

The main purpose of the current study was to explore the influence of RAEs throughout the Basketball England Talent Pathway according to gender. Key aims included the analysis of: (a) the BQ distributions of the Regional Talent Hubs (i.e., under-12 to under-U15) as an entry level into the Basketball England Talent Pathway, (b) the BQ distributions of the England National Youth Teams (i.e., under-16, under-18, and under-20), (c) the BQ distributions of those players who successfully transitioned from England National Youth Teams to the England National Senior Teams, and (d) the BQ distribution differences of the England National Youth Teams and England National Senior Teams based on the average number of minutes played. Key findings revealed RAEs were prevalent across the Regional Talent Hubs in both males and females, whereby BQ1s were up to ten times more likely to be selected compared with BQ4s. However, the BQ distributions across the England National Youth Teams were only significant in males. When exploring the youth- to senior-level transitions and the average number of minutes, the results show no differences between the BQ distributions in both males and females. In sum, the results of this study help to better understand the potential mechanisms of RAEs, as well as providing the impetus to explore more equitable selection and competition structures within the England Basketball Talent Pathway. As such, this discussion will attempt to offer considerations to reflect upon when designing implementing, and evaluating organisational structures in youth basketball, including: (a) gender, (b) age and competition levels, (c) youth- to senior-level transitions and playing time, and (d) proposing potential relative solutions and alternative group banding strategies.

According to gender, the impact of RAEs was greater in males compared to females, particularly in the England National Youth Teams and England National Senior Teams. In this regard, several studies revealed stronger RAEs in male youth sports compared to females [33,46]. The latest Sport England data show 26% of people aged 16+ years who play basketball regularly are female. Thus, similar to other sport contexts, English basketball comprises of lower participation in females compared to males [41]. As a result, the number of English teams and leagues is also lower (males = 99 teams, 9 leagues; females = 26 teams, 2 leagues), while the male top league is professional (i.e., players are salaried full-time) whereas the female top league is not. Therefore, a lower number of active players [13], and a lower depth of competition [47] could initially explain why these results showed a weaker prevalence of RAEs in females. Moreover, diverse developmental dynamics at puberty [48], an accelerated stabilisation of conditional-biological differences [32], and a variation in game demands [49] may have also contributed to weaker RAEs in females. Notwithstanding, RAEs in females are present in the Regional Talent Hubs. Accordingly, Smith et al. [13] revealed a higher relative age magnitude in female sports contexts among players aged 12 to 14 years. Furthermore, Delorme and Raspaud [21] observed significant RAEs in all youth categories of French female basketball players aged from 7 to 17 years, which appears more pronounced during puberty. These findings demonstrate that the current understanding of the mechanisms that explain RAEs in female players still needs to be improved.

The presence of RAEs decreases along the England Basketball Talent Pathway, which coincides with an increase in age and competition level. As in other talent identification and development systems (e.g., [50]), the physical and anthropometric advantages of relatively older players tend to reduce as the chronological age of the player increases. Thus, the selection processes may gradually become less affected by RAEs to the detriment of other factors, such as sport-specific skill level [51]. Nevertheless, and especially in basketball, factors such as height [25] or years at peak high velocity [52] are considered determinants in participation and competition performance, favouring relatively older players due to possible greater maturational development. On the other hand, however, it seems that a larger pool of potentially eligible basketball players at the senior levels would allow the recruitment methodology to solve itself [53]. Furthermore, the selection and re-selection processes at high-performance levels (e.g., talent pathways) have undergone significant changes in recent years, balancing the presence of relatively older and young players (e.g., [20,54]). In addition, Kalén et al. [30] demonstrated that relatively young players were 20–25% more likely to be reselected until age 20 than relatively older players due to initial selection age and long-term performance. Overall, it appears RAEs reduce with age and competition levels in high-performance basketball settings across both genders. Moving forward, selection processes should aim to recruit players based on their potential to achieve expertise in the long-term rather than focusing on individual and collective performance levels in the short-term.

Given the number of studies analysing the relative age phenomena in basketball, the information focused on the consequences of (bi) annual age group selection and performance is limited. Key findings showed the overrepresentation of relatively older players at youth levels did not translate into RAEs at senior levels or more minutes played for these players. More specialised sport contexts [55] and higher training levels [56] would imply an adjustment in the performance of players participating in talent pathways. Thus, players born at the end of the selection year would have the opportunity to overcome initial disadvantages (e.g., poorer access to training facilities, less expert coach support, and lower playing time among quality players), which may help them to acquire superior sport-specific skills compared with those born at the beginning of the selection year [39]. This reversal effect of relative age [37] or underdog hypothesis [38] may subsequently lead to higher performance levels for relatively younger players in the long-term. For instance, psychological characteristics, such as resilience, would help to reduce the differences caused by RAEs. Moreover, experiencing more stressful [57] or traumatic situations [58], greater effort in the learning process [59], training in pressurised conditions [60], and playing matches with higher levels of achievement [61] are factors that are often present in the sport transition processes of relatively younger players. Indeed, these experiences could enhance their performance capabilities in the long-term and, thus, increase the increased likelihood of successfully transitioning to senior levels or the number of minutes played. Furthermore, relatively younger players could suffer fewer injuries due to a low exposure to competition at formative ages, which could reduce their dropout rate and allow them to reach high-performance levels in the long-term [62]. On the other hand, however, those relatively younger players who are selected may be advanced in their maturation status and, thus, may have the physical skill set required in order to compete with their relatively older peers (e.g., [63]). Indeed, this may be why minutes played across the BQs were equally distributed. Overall, this study provides a relevant contribution in guiding the actions of coaches, clubs, and governing bodies to mitigate age unbalances in order to reduce the influence of RAEs. Further research is required to better understand the impact of RAEs on long-term performance, participation, and personal development outcomes to substantiate these suggestions.

### 4.1. Practical Implications and Future Research

Since there appears to be pronounced RAEs throughout the Basketball England Talent Pathway, it is important to consider possible relative age solutions and offer directions for future research. In the context of soccer, Mann and van Ginneken [64] designed an *age-ordered shirt numbering system* by providing stakeholders with the knowledge that the numbers on individuals’ playing shirts corresponded with relative age. Bennett et al. [65] recommended a *selection quota*, whereby stakeholders endorse policies that ensure clubs select a minimum number of players from each BQ. Tribolet et al. [66] proposed *avoiding early deselection* by encouraging stakeholders to avoid releasing players at young ages to ensure they have continued exposure to practice, competition, and resources without the option of being deselected. Romann et al. [67] suggested clubs and governing bodies should *delay the selection process* preferentially until post-maturation in order to make more fair and accurate decisions based on potential and negate the possible drawbacks of early specialisation. Grossmann and Lames [68] advised governing bodies to include the relative age phenomena into *coach education*, with the purpose of enhancing knowledge and understanding of RAEs as part of coaches’ formal coaching courses and/or training. Kelly et al., [39] conceptualised a *flexible chronological approach*, whereby early birth quartiles (i.e., BQ1s) and late birth quartiles (i.e., BQ4s) should be offered the opportunity to “play-up” (e.g., [69,70]) and “play-down” annual age groups, respectively. Kelly et al. [54] introduced birthday-banding, in which young athletes move up to their next birthdate group on their birthday with the aim to remove particular selection time points and specific chronological age groups. However, despite this range of possible relative age solutions, they are yet to be empirically evaluated in a basketball setting. As such, future research is required to explore the practical implications of these strategies within a youth basketball context.

The literature on alternative grouping strategies to moderate RAEs is limited when compared with the body of research demonstrating its prevalence. Moreover, where proposed grouping strategies have been suggested, little evidence has documented their effectiveness or directly implemented those strategies [71]. A useful strategy that may be utilised in basketball could be from the organisational policies incorporated in youth American football. More specifically, contrary to many other youth team sports, previous research has identified no RAEs in American football, which may lend credibility to the *age and anthropometric bandings* that are often employed to group their players (e.g., [72,73]). Another recent grouping approach that has produced promising results in youth soccer is *bio-banding*, which groups young players based on anthropometric and maturational status (see [74]). For instance, during their maturity-matched soccer competition, Bradley et al. [75] demonstrated how later-maturing players believed the bio-banded games offered greater opportunities to express themselves, adopt positions of leadership, and have a more important influence on gameplay. In comparison, early maturing players believed the bio-banded games were more physically and technically challenging. Both age and anthropometric and biological bands appear to systematically address one of the key mechanisms of RAEs, whereby relatively older athletes may have an advanced maturity status [14]. However, it is important to consider how these may look in the context of youth basketball, while the interaction effect of relative age and maturational status requires further study. Indeed, current studies have primarily focused their attention on academy soccer, thus, it is difficult to fully interpret how it will be conveyed within a basketball setting that is comprised of diverse talent development systems. Overall, although these banding approaches remain unproven in their impact on RAEs, an introduction to grouping players by height, weight, and/or some maturational variables may prove beneficial in moderating RAEs in youth basketball and, thus, warrants further research.

### 4.2. Limitations

It is important to consider the limitations of this study when interpreting its findings. First, playing position was not included because these data were not available. Previous research into playing position in basketball has shown a greater prevalence of RAEs in the guard position, whereas the centre position appears less affected [31]. Thus, it is important to consider playing position in future research to better understand who is more vulnerable to RAEs throughout respective talent pathways. Second, only one appearance for the England National Youth Teams and the England Senior National Teams were required to be included in this study. Since some players may have competed in considerably more games at both these levels, performance outcomes and career duration should be considered in future studies to examine the implications of RAEs on long-term development outcomes. Lastly, only within-1-year effects were explored based on annual competition cycles (i.e., BQ1 to BQ4), whereas constituent year effects based on biannual cycles (i.e., BQ1 to BQ8) as the national youth competition is organised were not measured. Previous research has shown how constituent year effects can impact opportunities to be selected into talent development pathways in basketball [22], thus, further study examining the impact of biannual age grouping is warranted.

## 5. Conclusions

It is evident that there is a complicated relationship between the BQ a player is born in, their opportunities to be selected into the Basketball England Talent Pathway, and their likelihood of successfully transitioning to senior levels. Key findings showed how RAEs were prevalent across the Regional Talent Hubs in both males and females, although they were only significant across the England National Youth Teams in males. When exploring the youth- to senior-level transitions and the average number of minutes played, the results show no differences between the BQ distributions in both males and females, suggesting possible reversal or underdog effects. Since there appears to be RAEs throughout the Basketball England Talent Pathway, researchers and practitioners are encouraged to work collaboratively to design, implement, and evaluate relative age solutions and alternative grouping strategies to create more equitable opportunities in youth basketball.

## Figures and Tables

**Table 1:** BQ distributions compared with National Norms (ONS, 2015) with chi-square analysis and BQ1 vs. BQ4 analysis.

Pathway	Cohort	BQ1	BQ2	BQ3	BQ4	Total	χ^2^ (df = 3)	*p*	Cramer’s V	BQ1 vs. BQ4 OR (95% CI)
Regional Talent Hubs	Male	40	28	16	4	88	32.338	**<0.001**	0.43	**9.98 (3.05; 32.59)**
	Female	38	29	14	14	95	17.365	**<0.001**	0.3	**2.27 (1.18; 6.23)**
	Combined	78	57	30	18	183	47.052	**<0.001**	0.36	**4.33 (2.25; 8.32)**
England National Youth Teams	Male	129	101	65	42	337	52.105	**<0.001**	0.28	**3.07 (1.94; 4.85)**
	Female	51	62	44	43	200	5.264	0.153	0.11	1.18 (0.64; 2.08)
	Combined	180	163	109	85	537	44.858	**<0.001**	0.2	**2.11 (1.49; 3.00)**
England National Senior Teams	Male	6	9	7	3	25	3.264	0.35	0.26	1.99 (0.34; 11.71)
	Female	5	5	6	3	19	1.095	0.778	0.17	1.66 (0.14; 4.59)
	Combined	11	14	13	6	44	3.806	0.283	0.21	1.83 (0.92; 6.68)

## Data Availability

Descriptive data is presented in Table 1.
